# Spatial and Environmental Variation of the Human Hair Microbiota

**DOI:** 10.1038/s41598-018-27100-1

**Published:** 2018-06-13

**Authors:** Lauren Brinkac, Thomas H. Clarke, Harinder Singh, Chris Greco, Andres Gomez, Manolito G. Torralba, Bryan Frank, Karen E. Nelson

**Affiliations:** 1 J. Craig Venter Institute, 9605 Medical Center Drive, Suite #150, Rockville, MD 20850 USA; 2 J. Craig Venter Institute, 4120 Capricorn Lane, La Jolla, CA 92037 USA; 3 Present Address: Department of Animal Science, University of Minnesota-Twin Cities, St Paul, MN 55108 USA

## Abstract

The skin is a complex living ecosystem harboring diverse microbial communities. Its highly variable properties and influence of intrinsic and extrinsic factors creates unique microenvironments where niche-specific microbes thrive. As part of the skin, hair supports its own microbial habitat that is also intra and inter-personal variable. This little explored substrate has significant potential in forensics microbiome research due to the unique signatures that are available on an individual. To further investigate this, we explored the hair microbiota from scalp and pubic regions in healthy adults to investigate how the hair shaft microenvironment varies microbially. Our results suggest that there are distinct differences between the microbial communities identified on hair shafts originating from different parts of the body. The taxonomic composition of the communities from different hair sources are most reminiscent of those identified from their associated cutaneous region. We further demonstrate that the hair microbiota varies by geographical origin and has the potential to be used to predict the source location of the hair.

## Introduction

The skin, our frontline defense against environmental antagonists, supports a living ecosystem of diverse habitats colonized by a range of microbes^1,2^. Microbial colonization is driven by the physiological and topological variation of the skin, contributing to distinct ecological niches and supporting complex microbial communities^2–5^. Cutaneous appendages, such as hair follicles and sebaceous and sweat glands, comprise sub-habitats that are associated with their own unique microbial species^6,7^. Most numerous on the face and scalp, the anoxic microenvironment of sebaceous glands support the growth of lipophilic bacteria such as *Propionibacterium* spp.^7^, whereas *Staphylococcus* and *Corynebacterium* spp. generally colonize skin regions associated with moist environments such as that of the inguinal crease^3^. Dry skin exhibits the greatest diversity with variable populations and abundances among the Actinobacteria, Proteobacteria, Firmicutes and Bacteriodetes phyla^3^. Influenced by both endogenous host (i.e., age, ethnicity etc.) and exogenous environmental (i.e., diet, geography, etc.) factors, the skin is a highly dynamic environment. Variations in skin properties will thus select for differences in microbial communities^8–11^, as seen in the microbiota of other anatomical areas^12–14^. Yet despite these variations, the skin microbiota remains relatively stable over time^4,15,16^.

As an outgrowth of the skin and part of the pilosebaceous unit, the hair shaft is also a likely source of colonizing bacteria. The microbiota of hair shafts originating from the scalp and pubic region are distinguishable^17^. Additionally, based on the relative abundance of Lactobacillaeae observed in the pubic hair microbiota, pubic hair samples can be discriminated by sex^17,18^. The abundance of Bifidobacteriales and Bacillales was also found to significantly vary between males and females, and the taxonomic distribution of the microbiota found on pubic hairs can be used to differentiate specific individuals^18^.

In this study, we explored the microbiota of scalp and pubic hair in healthy adults to investigate how these microecosystems vary across body sites and between individuals in different geographical locations. Results show significant compositional differences between hair shafts originating from scalp and pubic areas with the microbiota at each site mostly resembling microbial communities associated with adjacent cutaneous regions. We further show that variations in the hair shaft microbiota may be predictive of geographical origin of a sample.

## Methods

### Ethics Statement

The study was approved by the Institutional Review Board at the J. Craig Venter Institute (JCVI) (#2016-238), and all methods were performed in accordance with relevant guidelines and regulations. Written informed consent was obtained from all participants prior to sample collection.

### Cohort description and sample collection

Hair samples derived from scalp and pubic areas were collected from adults residing in Maryland (MD, n = 8) and California (CA, n = 8). Additionally, scalp hairs were collected from adults residing in Virginia (VA, n = 5). Both males and females from diverse ethnicities were recruited for this study. Samples were self-collected by participants over one week during late winter of 2016. Each individual provided multiple hairs, for a total of 42 and 32 hair samples from scalp and pubis respectively. The hair collection protocol was as described in Tridico *et al*.^17^. Shaft hair samples were self-collected at the same body location. Scalp hair was collected from behind the right ear, near the right retroauricular crease, and pubic hair was collected from their right pubis, near the right inguinal crease. Participants clipped rather than plucked hair to distinguish the hair shaft from the follicle. Prior to DNA extraction, hair length was measured and classified as short (<2 cm), medium (2–4 cm) or long (>4 cm).

### Sample preparation and DNA extraction

Hair samples were resuspended in 1200 ul of lysis buffer (20 mM Tris-Cl, pH 8.0, 2 mM EDTA, 1.2% Triton X-100) in preparation for DNA extraction. DNA from hair samples was extracted using enzymatic lysis; 200 mg/ml lysozyme (Sigma/Aldrich, St Louis, MO) and 20 mg/ml proteinase K (Life Technologies, Carlsbad, CA), followed by phenol chloroform isoamyl alcohol extraction and ethanol precipitation. Residual PCR inhibitors were removed using the MOBio Powerclean kit (MOBio Labs, Carlsbad, CA). DNA was quantified using fluorometric methods (SybrGold, ThermoFisher, Waltham, MA) prior to downstream applications.

### 16S rRNA gene V4 sequencing

Microbiota profiling was performed targeting the V4 region of the 16S rRNA gene. 16S rRNA gene amplification in each sample was performed using adaptor and barcode ligated V4 specific primers so that sequences from each sample in the library were identified with unique barcode indices. Mock community DNA was included in the library preparation step as described previously in Kozich *et al*.^19^. The mock community serves as a control for contaminants as well as a tool to ensure reproducibility and quality sequence reads, indicating the presence of unexpected spurious operational taxonomic units (OTUs). In addition, PhiX DNA was spiked into all sequencing runs as an integral control for sequencing. A high % of PhiX spike in (10–20%) adds diversity to 16S rRNA gene runs and improves quality. Amplicon from extraction controls and no template controls was also included to determine if any contamination occurred during DNA extraction or during the library prep stage. 16S rRNA gene libraries were analyzed on the High sensitivity DNA chip (Agilent) to ensure that libraries were free of adapter dimers contaminants and that they are appropriately sized for the platform. 16S rRNA gene libraries were sequenced using V2 chemistry 2 × 250 bp format on Illumina MiSEQ (Illumina Inc, La Jolla, CA) using standard manufacturer’s specifications. QC analysis was performed after each sequencing run where the % reads >= Q30, passing filter clusters and yield/sample were monitored.

### 16S rRNA gene quantification

To determine the absolute quantification of the bacterial biomass in each sample, quantitative real-time polymerase chain reaction (qPCR) was performed using 1 µl of each sample (20 uL total reaction volume) with LightCycler® 480 SYBR Green I Master (Roche Diagnostics, Rotkreuz, Switzerland). Reactions were performed in duplicate using the LightCycler® 480 (Roche Diagnostics). The following amplification protocol was used: 60 cycles each of 95 °C for 10 sec, 60 °C for 10 sec, and 72 °C for 30 sec with single acquisition, using 16S rRNA V4 primers^19^ at a final concentration of 200 nM. *Streptococcus pneumoniae* serotype 4 strain TIGR4 genomic DNA (NC_003028) was used as the positive control, and a melt curve was performed to confirm specificity of the primers for the target.

### 16S rRNA gene sequence data analysis

Sequence reads from the 74 hair samples obtained plus 2 negative controls were processed using an in-house 16S rRNA gene data analysis pipeline. Operational taxonomic units (OTUs) were generated using the default parameters in UPARSE^20^ and taxonomies were assigned to these OTUs with mothur^21^ using 123 version of the SILVA 16S rRNA gene database^22^ as the reference database. Samples with more than 500 reads (65 samples) were further considered for downstream analysis. OTU count tables were normalized to relative abundances of reads mapping to different taxa at all taxonomic levels using the R-package Phyloseq^23^.

### Statistical analysis

Non-metric multidimensional scaling (NMDS) graphs were generated using the Phyloseq R-package, while the permutational multivariate analysis of variance (PERMANOVA) calculations were performed to detect statistical significance using the VEGAN R-package using Bray-Curtis dissimilarity matrix^24^. To detect differential abundances in the hair microbiota at the genus level, phyloseq data was converted into a DESeq2 object using the phyloseq_to_deseq2 function, and DESeq2 package version 1.12.3 in R was used^25^ for differential abundance. DESeq2, using a local fit type to estimate dispersions, was used for its multiple testing adjustment applying Benjamini & Hochberg False Discovery Rate^26^. The p-value cutoff for the selection of significant OTUs is 0.05 after false discovery rate (FDR) adjustment for multiple comparisons. Random Forest algorithm implemented in R was used to perform classification of the MD vs CA samples.

### Availability of data and materials

Raw datasets and associated metadata generated and analyzed as part of this study are available in the NCBI SRA database under the accession number: SRP149455 as part of the NCBI Bioproject PRJNA417700. Processed datasets can be analyzed in comparison with other publicly available human microbiota data through the Forensic Microbiome Database (FMD) http://fmd.jcvi.org/.

The processed read sequences analyzed from the HMP are available in the HMP Data Analysis and Coordination Center, http://hmpdacc.org/HM16STR/^1^.

## Results

### Microbial biomass variability and alpha diversity

**Figure 1:**
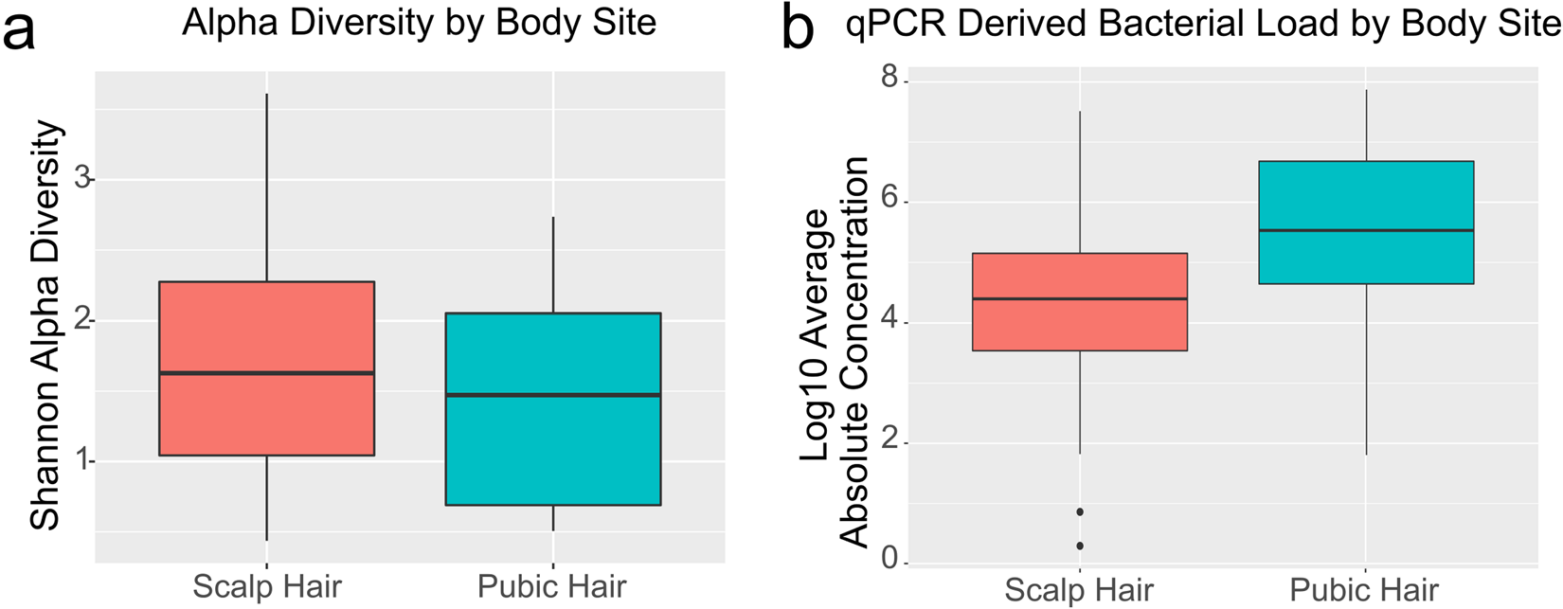
The 16S rRNA Gene Sequencing Biomass and Alpha Diversity by Collection Site. (**a**) Alpha diversity (Shannon index) in hair shaft samples taken from the scalp and pubic hairs (>500 reads). (**b**) Microbial biomass of hair shaft samples taken from the scalp and pubis.

Molecular and statistical analysis of the hair microbiota revealed 16S rRNA gene bacterial biomass and alpha diversity differences between hair originating from different body sites. Pubic hair has significantly more biomass than scalp hair (Pubic mean = 7.23e6; Scalp mean = 2.47e5; 2-sided t-test, p = 0.044), and there is a significant difference in alpha diversity (Shannon index) of the two hair types (ANOVA p = 0.02), with pubic hair showing lower alpha diversity than scalp hair (Fig. 1). Considering hair length, there is no significant difference in the bacterial biomass of scalp or pubic hair of varying hair lengths. However, longer scalp hairs show significant greater alpha diversity (Shannon index) than shorter hairs (ANOVA p = 0.004), though this is not seen with pubic hairs (ANOVA p = 0.7) (Supplementary Fig. S1). Samples generated from multiple hairs (n = 10) produced more biomass compared to using a single hair, and the amount did positively correlate with number of hairs for both pubic (r = 0.154, p = 0.0151) and scalp hairs (r = 0.52, p = 5.12e-08), but not with higher alpha diversity (Supplementary Fig. S2).

### Taxonomic composition and structure

**Figure 2:**
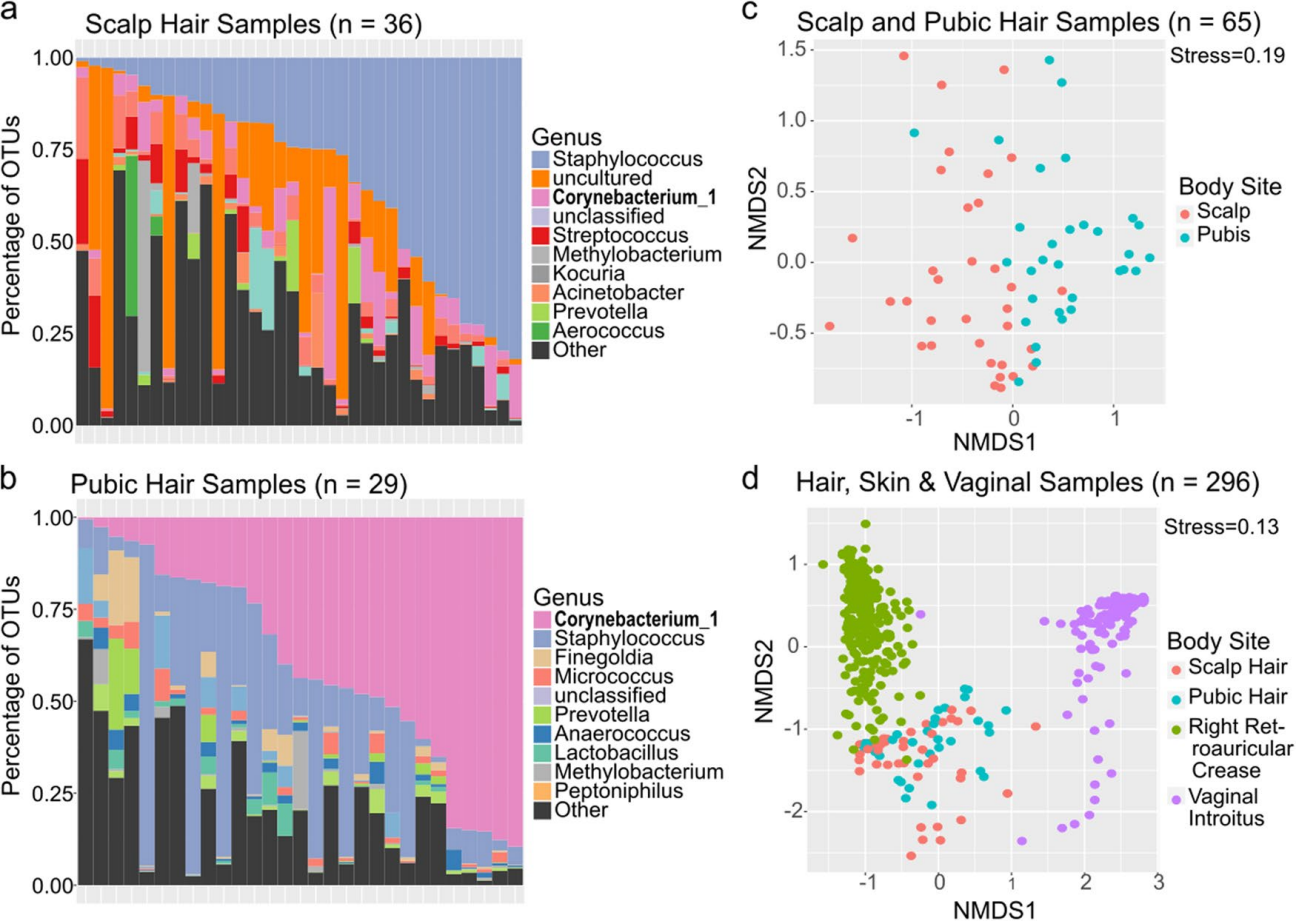
Taxa Distribution of Hair Shaft Samples to Corresponding Human Microbiome Project (HMP) Sites. Taxonomic profiles (genus level) of scalp hair shaft samples (**a**) and pubic hair shaft samples (**b**) with significant differentially abundant genera by DESeq2 in bold. Weighted NMDS plot of microbiome composition differences (OTU level) between the scalp and the pubic hair samples (**c**) NMDS plot between the scalp and the pubic hair samples and HMP samples from the right retroauricular crease and the vaginal introitus body sites (**d**).

Hair shafts from both scalp and pubis areas showed a similar taxonomic profile but in different abundances (Fig. 2a,b). In both body sites, a majority of the sequences map to four dominant taxa (pubic hair = 69%, scalp hair = 61%). The top four taxa identified among the scalp hair microbiota is *Staphylococcus*, uncultured, *Corynebacterium*, and unclassified, while the top four taxa identified among the public hair microbiota are *Corynebacterium*, *Staphylococcus*, *Finegoldia*, and *Micrococcus*. The genus *Staphylococcus* is similarly abundant in both sites (24% in pubic hair and 32% in scalp), while uncultured bacteria is more abundant in scalp than in the pubic hair respectively (18% vs 2%), and *Corynebacterium* is more abundant in the latter (7% vs 40%) (DESeq2, adjusted p = 6.38e-4). Of the scalp hair genera not classified (comprising 10 OTUs (10%)), nine have a taxonomic rank at the phyla level. An NMDS ordination plot showed compositional differences between the microbiota of the two hair types that were statistically different based on Bray-Curtis distance (PERMANOVA, r^2^ = 0.13, p = 0.001) (Fig. 2c).

Our findings also show taxonomic patterns similar to previous hair and skin microbiota studies, with some distinct differences emerging. Overall, taxonomic patterns between the hair samples were more similar to each other than to the skin, right retroauricular crease, or vaginal samples, vaginal introitus, from the Human Microbiome Project (HMP)^1^, with the vaginal samples being most distinct (Fig. 2d). Previous work on scalp and pubic hair also found that *Corynebacterium* is either the most or second most abundant genus^17^. However, other common genera found in the hair microbiota like *Staphylococcus* and *Streptococcus* are not observed^17^, but are relatively common in the skin microbiota described by the HMP^1^ or Grice *et al*.^3^. Likewise, we found *Anaerococcus*, an abundant genus in both the scalp and pubic hair transcriptomes^17^, at lower abundance (1.6% in pubic hairs and 0.7% in scalp hairs). Pubic hair shows the largest disagreement compared with other studies. For instance, the most predominant taxa observed in HMP vaginal introitus samples^1^, *Lactobacillus* represents the most abundant species in female pubic hair microbiota^17,18^, whereas in our study *Lactobacillus* was found at low abundance (1.6%). Sex and ethnic data was not collected from participants, therefore this observation of low abundance could be skewed due to the combined analysis of pubic hair samples regardless of sex or it could be reflective of the inherent vaginal microbiota variability of women from different ethnicities^14,27,28^. Noticeably absent from our scalp hair samples is the genera *Propionibacterium*, which has been reported as the most abundant species in three scalp skin microbiota studies^29–31^ as well as in right retroauricular crease skin samples from the HMP^1^. This absence could be attributed to a niche preference of *Propionibacterium* which thrives in an anaerobic environment such as that of the hair follicle where sebum serves as a nutritional source^32^.

### Geographical variability

**Figure 3:**
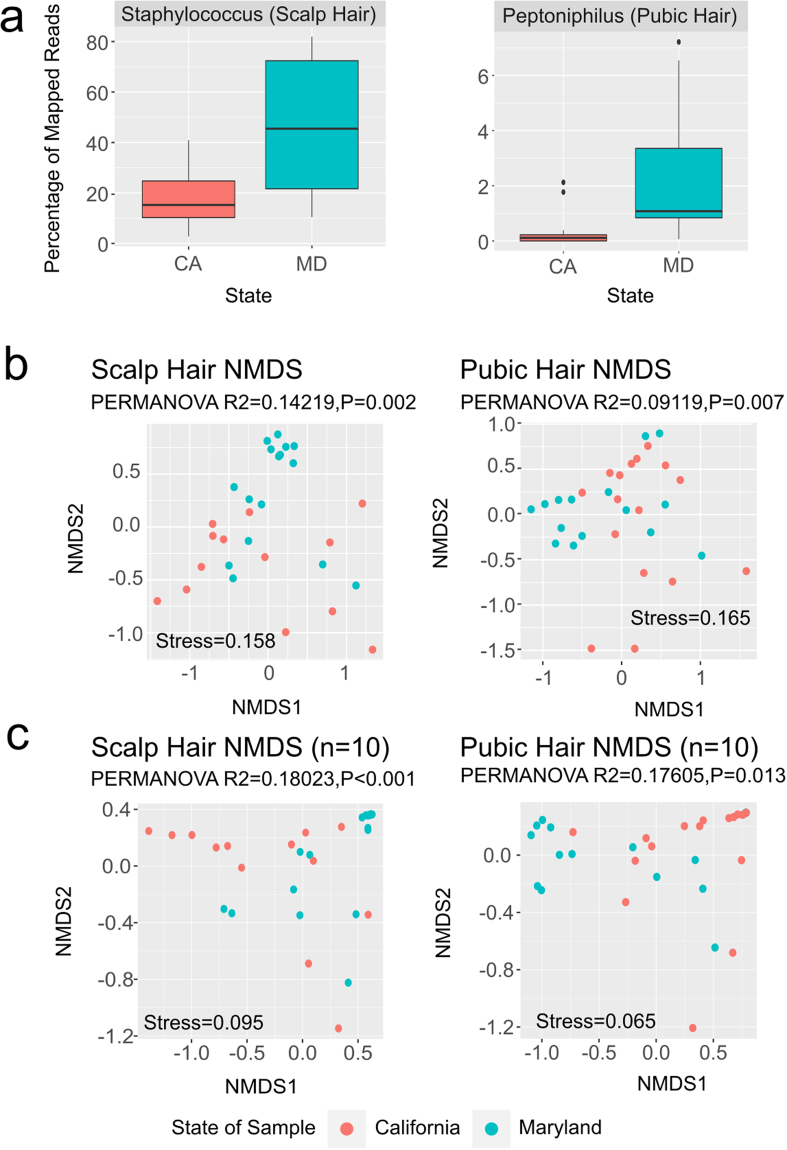
Hair microbiota differences according to hair type (scalp and pubic) and geographical location (CA and MD). (**a**) Differentially abundant taxa for scalp (*Staphylococcus*) and pubic (*Peptoniphillus*) hair between hairs collected in La Jolla, CA and Rockville, MD. (**b**) Weighted NMDS plots showing scalp and pubic hair microbiome composition differences between subjects from La Jolla and Rockville. (**c**) Weighted NMDS plots based on the most discriminant 10 taxa selected by mean decrease GINI from the Random Forest classification analyses from each of scalp and pubic hair.

Microbial communities differ in composition and function across different geographical locations. Random Forest analysis was performed to determine if differences in the hair microbiota are associated with geography. We performed DESeq2^25^ differential abundance testing on scalp and pubic hair and observed *Peptoniphilus* and *Staphylococcus* as differentially abundant at FDR 0.05 when comparing MD and CA samples based on pubic and scalp hair samples respectively (Fig. 3a). Even though the weighted NMDS plots do not show significant clustering based on geographical location in each hair type (Fig. 3b), we achieved 17.24% out-of-bag (OOB) error rate and 0.93 Area Under the Curve (AUC) using Random Forests showing moderate classification values for MD vs. CA using scalp hair samples. In the case of pubic hair samples, we obtained higher OOB error rate 22.58% and lower AUC 0.82, suggesting that scalp hair has higher geolocation prediction power as compared to pubic hairs. Likewise, using only the 10 most distinct genera as identified by mean decrease GINI from the Random Forest analyses (Supplementary Tables S1 and S2) increases the geolocation power of the hair samples as compared to using all the taxa **(**Fig. 3c) in both scalp and pubic hair.

## Discussion

These results offer a novel perspective to a study area of the human microbiota that has been traditionally neglected: the microbiota of human hair. Our study confirms that human hair shafts harbor unique bacterial communities, distinctive from that of the hair follicle and more reminiscent of its associated cutaneous region. *Staphylococcus*, a common genera found in the skin^1,3^, was also found to be abundant in hair. However, *Propionibacterium,* a predominant bacterium that colonizes the skin and hair follicles^7^, is noticeably absent in this dataset. The hair shaft environment may be unfavorable for growth of *Propionibacterium* which prefer low oxygen levels and high sebum content as that of the hair follicle^32^. This finding is consistent with other hair studies where the hair was cut rather than plucked to differentiate the hair shaft from the follicle^17,18^. Like the skin, the hair microbiota varies regionally on the body with bacterial composition and structure differing between varying hair environments. *Corynebacterium*, a differentially abundant taxa identified in pubic hair prefers moist environments such as that of the groin^3^. This result differs from previously published hair studies where *Lactobacillus* is the predominant genus identified in female pubic hair samples^17,18^, highlighting the importance of considering sex and ethnicity in future hair microbiota research.

Variable characteristics of hair, such as hair length, are one of many influential factors that may affect the intra-personal variability of hair microbial communities. Our study suggests that longer scalp hair (>4 cm) shows significantly greater alpha diversity than shorter hairs (<4 cm), an observation not found with pubic hairs. Thus, from a sampling perspective, actual hair microbiota variability may be confounded by technical sources of variation. This could be particularly challenging for forensic applications of the scalp hair microbiota, but shows promise of the pubic hair microbiota as suggested by other studies^17,18^.

Human hair shafts also show distinct geographic variation in the microbial communities. Comparisons between the hairs collected in CA and MD show scalp hair having greater potential to predict geolocation than pubic hair, which is of critical relevance for forensic applications. However, as human scalp hair generally interacts with the environment more so than pubic hair, it is possible that any geographic signature on the scalp hair will be comparatively transient, or intrinsically linked to environmental or lifestyle factors. This difference highlights the value of hair microbiota as biomedical or forensic tools, with scalp and pubic hair being relevant in specific scenarios (e.g. geolocation, gender/individual identification or biomarker detection). Increasing sample sizes and performing longitudinal studies would help further clarify the usefulness of both types of hair as an indicator of forensic information.

## Electronic supplementary material

Supplementary Data
