# Supplementary material for: Spatial and Environmental Variation of the Human Hair Microbiota
Source: Sci Rep. 2018 Jun 13;8:9017. doi: 10.1038/s41598-018-27100-1 (PMC5997989; doi:10.1038/s41598-018-27100-1)
Supplement: Supplementary file 1 — Supplementary Data [file 41598_2018_27100_MOESM1_ESM.pdf]

Spatial and Environmental Variation of the Human Hair Microbiota

Supplemental Material

Lauren Brinkac[1]*, Thomas H Clarke[1], Harinder Singh[1], Chris Greco[1], Andres Gomez[2,3], Manolito G. Torralba[2], Bryan Frank[1] and Karen E. Nelson[1]

[1] J. Craig Venter Institute, 9605 Medical Center Drive, Suite #150, Rockville, MD 20850, USA
[2] J. Craig Venter Institute, 4120 Capricorn Lane, La Jolla, CA 92037, USA
[3] Current Address: Department of Animal Science, University of Minnesota-Twin Cities, St Paul, MN 55108, USA

*Corresponding Author
lbrinkac@jcvi.org
Tel: +1 301-795-7867

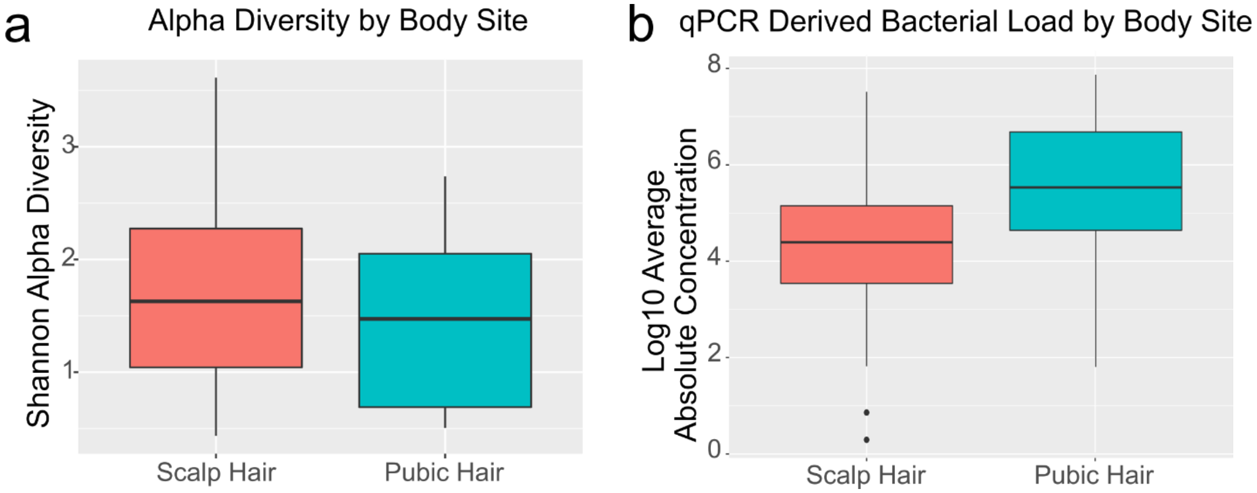

Figure S1. The 16S rRNA Gene Sequencing Biomass and Alpha Diversity by Hair Length. (a) Alpha diversity (Shannon index) in single hair shaft samples from the scalp and pubis (> 500 reads) that were classified as short (<2cm), medium length (2-4 cm) or long (>4cm). Pairs of hair lengths having significantly different alpha diversity using Tukey test adjusted p-value < 0.05 are shown by an asterisked bracket. **(b)** Microbial biomass of single hair shaft samples taken from the scalp and pubis also divided by length into short, medium and long.

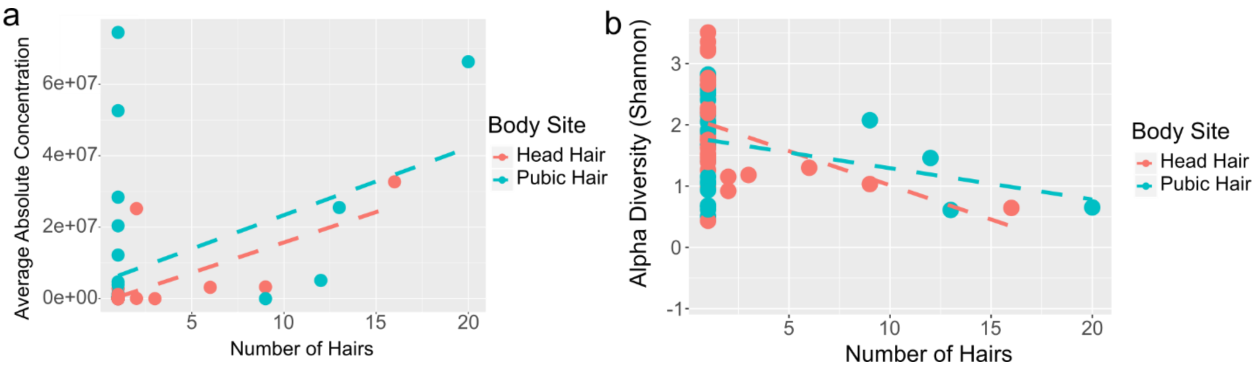

Figure S2. The 16S rRNA Gene Biomass and Alpha Diversity by Number of Hairs. (a) Alpha diversity (Shannon index) in hair shaft samples from the scalp and pubis (> 500 reads) and the number of hairs in the sample with the least-squares fitted line for both body sites. **(b)** Microbial biomass and the number of hairs from hair shaft samples taken from the scalp and pubis with the least-squares fitted line for both body sites.

10 Most Decrimintory OTUs by GINI for Scalp Hair Microbiomes (highlight = significant at 0.05 Q-Value)

OTU ID	Family	Genus	GINI Value	Mean California Percentage	Mean Maryland Percentage	Wilcox Test P-Value	Wilcox Test Q-Value
FMD000012	Micrococcaceae	Kocuria	0.68229092	0.965172048	3.712031944	0.147675469	0.593059349
FMD000001	Staphylococcaceae	Staphylococcus	0.60899489	17.39830359	46.89026728	0.001497821	0.01497821
FMD000004	Corynebacteriaceae	uncultured	0.59386349	32.86157393	6.858457685	0.016100109	0.128800875
FMD000003	Corynebacteriaceae	Corynebacterium_1	0.49250097	3.112580085	11.023426	0.003509965	0.031589689
FMD000074	Moraxellaceae	Acinetobacter	0.35488166	2.723354148	0.602103167	0.320434084	0.640868169
FMD000014	Moraxellaceae	Moraxella	0.35242271	0.920197984	0.649213354	0.085854457	0.515126745
FMD000018	Lactobacillaceae	Lactobacillus	0.32335105	0.70402919	0.226695976	0.175141202	0.593059349
FMD000049	Pseudomonadaceae	Pseudomonas	0.32184191	1.267137573	1.091791952	0.11861187	0.593059349
FMD000039	Neisseriaceae	Neisseria	0.26424117	0.900944934	0.963696306	0.019970304	0.139792131
FMD000067	Micrococcaceae	Rothia	0.24063822	1.688805547	0.080220311	0.336430223	0.640868169

10 Most Decrimintory OTUs by GINI for Pubic Hair Microbiomes (highlight = significant at 0.05 Q-Value)

OTU ID	Family	Genus	GINI Value	Mean California Percentage	Mean Maryland Percentage	Wilcox Test P-Value	Wilcox Test Q-Value
FMD000012	Micrococcaceae	Kocuria	0.99674481	1.038537447	1.281160985	0.01984543	0.138918012
FMD000001	Staphylococcaceae	Staphylococcus	0.55741406	25.95413832	21.79586357	0.281424733	0.281424733
FMD000003	Corynebacteriaceae	Corynebacterium_1	0.47987844	29.48752272	47.57175411	0.065509274	0.262037096
FMD000016	Family_XI	Anaerococcus	0.42085618	1.226276951	2.187217006	0.092833285	0.278499855
FMD000015	Family_XI	Peptoniphilus	0.38678974	0.340822354	2.348411613	0.000694675	0.006946754
FMD000043	Aerococcaceae	unclassified	0.33903751	0.093136837	0.918480872	0.034055457	0.20433274
FMD000009	Streptococcaceae	Streptococcus	0.32582809	1.719705581	0.862048876	0.103861227	0.278499855
FMD000011	Brevibacteriaceae	Brevibacterium	0.30669191	0.221613413	2.029729987	0.00308853	0.024708239
FMD000090	Actinomycetaceae	Mobiluncus	0.30129642	0	0.236306328	0.002737866	0.024640793
FMD000098	Veillonellaceae	Dialister	0.29091818	0.208532011	0.226934021	0.05007371	0.250368549
